# Cognitively healthy centenarians are genetically protected against Alzheimer's disease

**DOI:** 10.1002/alz.13810

**Published:** 2024-04-18

**Authors:** Niccolo’ Tesi, Sven van der Lee, Marc Hulsman, Natasja M. van Schoor, Martijn Huisman, Yolande Pijnenburg, Wiesje M. van der Flier, Marcel Reinders, Henne Holstege

**Affiliations:** ^1^ Delft Bioinformatics Lab Delft University of Technology Delft The Netherlands; ^2^ Department of Clinical Genetics Section Genomics of Neurodegenerative Diseases and Aging Vrije Universiteit Amsterdam, Amsterdam UMC Amsterdam The Netherlands; ^3^ Department of Neurology Alzheimer Center Amsterdam Amsterdam Neuroscience, Vrije Universiteit Amsterdam, Amsterdam UMC Amsterdam The Netherlands; ^4^ Department of Epidemiology and Data Sciences Amsterdam UMC location Vrije Universiteit Amsterdam Amsterdam The Netherlands; ^5^ Mental Health Program Amsterdam Public Health Research Institute Amsterdam The Netherlands

**Keywords:** Aging, Alzheimer's disease, Cognitively healthy centenarians, Endolysosomal, Genes, Genome‐wide association studies, Heritability, Immunity, Protection, Resilience

## Abstract

**BACKGROUND:**

Alzheimer's disease (AD) prevalence increases with age, yet a small fraction of the population reaches ages > 100 years without cognitive decline. We studied the genetic factors associated with such resilience against AD.

**METHODS:**

Genome‐wide association studies identified 86 single nucleotide polymorphisms (SNPs) associated with AD risk. We estimated SNP frequency in 2281 AD cases, 3165 age‐matched controls, and 346 cognitively healthy centenarians. We calculated a polygenic risk score (PRS) for each individual and investigated the functional properties of SNPs enriched/depleted in centenarians.

**RESULTS:**

Cognitively healthy centenarians were enriched with the protective alleles of the SNPs associated with AD risk. The protective effect concentrated on the alleles in/near *ANKH, GRN, TMEM106B, SORT1, PLCG2, RIN3*, and *APOE* genes. This translated to >5‐fold lower PRS in centenarians compared to AD cases (*P *= 7.69 × 10^−71^), and 2‐fold lower compared to age‐matched controls (*P *= 5.83 × 10^−17^).

**DISCUSSION:**

Maintaining cognitive health until extreme ages requires complex genetic protection against AD, which concentrates on the genes associated with the endolysosomal and immune systems.

**Highlights:**

- Cognitively healthy cent enarians are enriched with the protective alleles of genetic variants associated with Alzheimer's disease (AD).
- The protective effect is concentrated on variants involved in the immune and endolysosomal systems.
- Combining variants into a polygenic risk score (PRS) translated to > 5‐fold lower PRS in centenarians compared to AD cases, and ≈ 2‐fold lower compared to middle‐aged healthy controls.

## 1 BACKGROUND

The average human life expectancy continues to grow and by 2050 there will be 3.2 million centenarians in the world.^1^ At old ages, a major contributor to poor health is cognitive decline and dementia, of which Alzheimer's disease (AD) is the most common type.^2^, ^3^ However, AD is not an inevitable consequence of aging, as testified by a small proportion of the population that reaches at least 100 years while maintaining a high level of cognitive and physical functions.^4^, ^5^ This raises the question of whether these cognitively healthy centenarians have exceptional features that protect or delay the onset of dementia, and whether such mechanisms may be genetically encoded.

AD is a progressive disorder characterized by loss of cognitive functions, ultimately leading to loss of independence and death, for which an effective treatment is lacking.^3^, ^6^ The greatest risk factor for AD is age: the disease is rare at 60 years, and the incidence of AD reaches ≈ 40% per year at 100 years of age.^7^ Next to aging, heritability plays an important role that changes dramatically with age. While the heritability of AD with age at onset < 65 years is estimated to be 90% to 100%, mostly due to autosomal dominant or strong risk‐increasing genetic variants,^8^ it decreases to 60% to 80% for ages at AD onset of ≈ 75 years (determined by twin studies), based on a unique mix of rare and common risk factors, and further declines with later ages at AD onset.^9^ Approximately 30% of the genetic risk of AD is attributable to the ε4 allele of the apolipoprotein E (*APOE*) gene. Large collaborative genome‐wide association studies (GWAS) have collectively identified 86 single nucleotide polymorphisms (SNPs) that are associated with a slight modification of the risk of AD.^10^, ^11^

Intriguingly, the reverse is also true, as ≈ 60% of the chance to survive to 100 years in good cognitive health depends on inheriting favorable genetic factors,^12^ comprising a relative depletion of risk‐increasing variants and an enrichment of advantageous genetic variants that associate with a prolonged (brain) health.^13^, ^14^, ^15^ In fact, in 2018 we reported that the effect size of 29 SNPs that were associated with AD risk was increased on average 2‐fold when using cognitively healthy centenarians as controls rather than controls age‐matched with the AD cases.^16^ Consequently, cognitively healthy centenarians had a significantly lower polygenic risk score (PRS), compared to AD cases and age‐matched controls.

In the current study, we aimed to further expand on these findings by investigating the prevalence in cognitively healthy centenarians of the 86 SNPs that are currently associated with AD risk, based on the most recent GWAS for AD.^10^ We studied the effect of individual AD‐associated SNPs as well as their combined effect (PRS) on prolonged cognitive health. Furthermore, we identified risk‐increasing and protective SNPs that were, respectively, most depleted or enriched in a cohort of cognitively healthy centenarians, which allowed us to highlight the biological mechanisms most strongly involved with resilience against AD.

## 2 METHODS

### 2.1 Cohort description

We included 6747 individuals in this study. Of these, 2542 were AD cases, either clinically diagnosed with probable AD from the Amsterdam Dementia Cohort (ADC, *N* = 2060)^17^, ^18^, ^19^ or pathologically confirmed from the Netherlands Brain Bank (*N* = 482).^20^ The diagnosis of probable AD in the ADC cohort was based on the clinical criteria formulated by the National Institute of Neurological and Communicative Disorders and Stroke–Alzheimer's Disease and Related Disorders Association (NINCDS‐ADRDA) and based on the National Institute of Aging–Alzheimer Association (NIA‐AA). All subjects underwent a standard diagnostic assessment including neurological examination, blood tests, magnetic resonance imaging, electroencephalogram, and cerebrospinal fluid (CSF) analysis (available for most patients). Together, this diagnostic procedure reduces the chance of misdiagnosis.^17^ As age‐matched controls, we used (1) a sample of 1776 Dutch older adults from the Longitudinal Aging Study of Amsterdam (LASA),^21^ (2) a sample of 1524 older adults with subjective cognitive decline who visited the memory clinic of the Alzheimer Center Amsterdam and SCIENCe project and were labeled cognitively normal after the extensive examination,^17^ (3) a sample of 62 healthy controls from the Netherlands Brain Bank,^20^ (4) a sample of 196 individuals from the twin study,^22^ and (5) a sample of 85 older adults from the 100‐plus Study (partners of centenarians’ offspring).^5^ All age‐matched controls were cognitively healthy at the time of inclusion in this study. Individuals with subjective cognitive decline were followed over time in the SCIENCe project, and only individuals who did not convert to mild cognitive impairment or dementia during follow‐up were included in this study. Additional information about inclusion criteria for these cohorts is available in Supplementary Material. As alternative (extreme) healthy controls, we used 360 cognitively healthy centenarians from the 100‐plus Study cohort.^5^ This study includes Dutch‐speaking individuals who (1) can provide official evidence for being aged ≥ 100 years; (2) self‐report to be cognitively healthy, which is confirmed by a proxy; (3) consent to the donation of a blood sample; (4) consent to (at least) two home visits from a researcher including an interview and neuropsychological test battery.^5^ The medical ethics committee of the Amsterdam UMC approved all studies. All participants and/or their legal representatives provided written informed consent for participation in clinical and genetic studies.

### 2.2 Genotyping and imputation of 86 selected SNPs

We included 85 SNPs that were significantly associated with AD in the latest GWAS by Bellenguez et al.,^10^ plus SNP rs12459419 near *CD33* (Table S1 in supporting information).^23^, ^24^ After quality control and genotype imputation of the genetic data (see Supplementary Methods: Genotyping and Imputation), all individuals passed quality control. Before analysis, we excluded individuals with a family relation (identity‐by‐descent ≥ 0.2),^25^ and we kept only individuals of European ancestry (based on 1000Genomes clustering),^26^ leaving 2281 AD cases, 3165 age‐matched controls, and 346 cognitively healthy centenarians for the analyses.

### 2.3 Single variant analyses

As reference effect size for each SNP, we used the effect sizes resulting from the comparison of 39,106 clinically diagnosed AD cases and 401,577 age‐matched controls used in the discovery phase by Bellenguez et al. (Table S1).^10^ We excluded the proxy phenotypes which Bellenguez et al. included in their multi‐stage meta‐analysis, as these are based on paternal and maternal disease status rather than clinical diagnosis, which typically leads to a dilution of the SNP effect sizes. For each AD‐associated SNP, we calculated the change in effect size relative to the reference effect size comparing (1) AD cases versus cognitively healthy centenarians, (2) AD cases versus age‐matched controls, and (3) age‐matched controls versus cognitively healthy centenarians (see Supplementary Methods: Change in effect size). In a sensitivity analysis, we compared the frequency of each SNP between early‐onset (age at onset ≤ 65 years) and late‐onset AD cases, to highlight potential genetic modifiers.

**RESEARCH IN CONTEXT**

1. **Systematic review**: Genetic studies of Alzheimer's disease (AD) have identified common genetic variants that influence the risk of AD. By contrast, the genetic factors associated with long‐term resilience against AD are mostly elusive. We studied the genetic variants associated with AD in cognitively healthy centenarians, that is, individuals > 100 years with maintained cognitive health.
2. **Interpretation**: Cognitively healthy centenarians were enriched with the protective alleles of AD‐associated variants. The variants with the largest effect size in centenarians functionally map to endolysosomal and immunological/clearance mechanisms. A polygenic risk score combining all AD variants was > 5‐fold lower in the cognitive healthy centenarians compared to AD cases and almost 2‐fold lower compared to AD age‐matched controls.
3. **Future directions**: Our article highlights the importance of further investigation of protective genetic variants and their effects on maintaining health. The prioritization of the biological processes associated with the strongest protection pinpoints those mechanisms involved in the resilience against AD.

### 2.4 Polygenic risk score

We combined all 86 SNPs into a PRS, resembling an individual's net genetic risk of AD. As weights for the PRS, we conventionally used the effect sizes of the meta‐analysis including both clinically diagnosed AD cases and by‐proxy phenotypes, reflecting the final results of Bellenguez et al. (Table S1). Given the large effect size associated with the two *APOE* SNPs (rs429358 and rs7412), we calculated PRS including and excluding these two SNPs. We assessed the association between PRS and AD risk by comparing the scaled PRS distributions (μ = 0, σ = 1) between AD cases, age‐matched controls, and cognitively healthy centenarians, in a pairwise manner and splitting by sex. We also tested for any difference in the PRS between early‐onset AD and late‐onset AD samples. For the associations, we used logistic regression models adjusting for population stratification (PC1‐5). The resulting effect sizes (log of odds ratio) can be interpreted as the odds ratio difference per one standard deviation increase in the PRS, with the corresponding 95% confidence intervals.

### 2.5 The contribution of a centenarian

In genetic studies, the power to detect a significant SNP association is affected by both the effect size and the sample size, which includes the number of cases and controls in the comparison. This can be approximated using power analyses. The greater the effect size, the smaller the required number of cases and controls to achieve statistical significance in an association. Through a power analysis, we determined the potential additional statistical power offered by cognitively healthy centenarians compared to typical controls in a case‐control study of AD. To do so, for each SNP identified by Bellenguez et al. we calculated the number of normal (age‐matched) controls and cognitively healthy centenarians necessary to obtain 80% power to find a SNP association at *P* value = 0.05. We assumed (1) 8000 AD cases, (2) the minor allele frequency as reported in the reference GWAS (Table S1), and (3) the observed effect size from our comparisons (AD cases versus age‐matched controls, and AD cases versus cognitively healthy centenarians). Because the direction of effect must be consistent with the direction reported in Bellenguez et al. we excluded SNPs for which we observed an opposite direction of effect in both AD cases versus age‐matched controls and AD cases versus cognitively healthy centenarians. Then, for each SNP, we compared 8000 AD cases to 200 controls, and recursively increased the number of controls by 200 until a power of at least 80% was found or the number of controls was twice the number of AD cases (i.e., 16,000). When a SNP association reached at least 80% power, we regarded it as converging. The ratio between the number of age‐matched controls and cognitively healthy centenarians, for each SNP, indicates the increase in statistical power of a single centenarian relative to age‐matched controls. We simulated the analysis using several thresholds for the number of AD cases to use (2500, 5000, 8000, and 10,000) and found that after 8000 no additional SNPs converged.

### 2.6 In silico functional analysis

We investigated the biological pathways associated with the SNPs with the largest effect‐size differences between cognitively healthy centenarians and age‐matched controls. We selected SNPs for which, based on our power analysis, the number of cognitively healthy centenarians was at least half of the number of age‐matched controls to achieve the same power. For the functional analysis, we used the functional annotation section of snpXplorer web server with default settings.^27^ This tool performs (1) variant‐to‐gene mapping using integrating variant consequences (coding, intronic, intergenic) and quantitative trait loci (eQTLs and sQTLs), followed by (2) gene‐set enrichment analysis, and (3) clustering of the enriched terms.^27^ The clusters of enriched terms were compared to clusters obtained from a previous study including all AD‐associated SNPs based on the same method.^27^

### 2.7 Implementation

Quality control of the genotype data, population stratification analysis, and relatedness analyses were performed with PLINK (v1.90 and v2.0). Association analyses, downstream analyses, and plots were performed with R (v4.2). For the power analyses, we adapted the likelihood ratio test framework implemented in the R package genpwr.^28^ The scripts are publicly available at https://github.com/TesiNicco/Centenarians_AD.

## 3 RESULTS

### 3.1 Quality control of genetic data and SNPs

The mean age at study inclusion of the 2281 AD cases was 67.96 ± 9.84 (interquartile range [IQR] = [61–74], 55% females, of which 971 were early‐onset AD with age at diagnosis ≤ 65 years), the mean age of the 3165 age‐matched controls was 62.57 ± 8.66 (IQR = [57–66], 48% females), and the mean age of the 346 cognitively healthy centenarians was 101.05 ± 2.51 (IQR = [100–102], 71% females; Table 1, Figure S1 in supporting information). The median quality of the imputed SNPs was *r* ^2^ = 0.95 and ranged from 0.45 to 0.99 (Table S2 in Supplementary Tables); all imputed SNPs were included in the analyses.

**TABLE 1:** Population characteristics.

	*AD cases*	*Age‐matched controls*	*Cognitively healthy centenarians*
**Sample size**	2281	3165	346
**Age**	67.96 ± 9.84	62.57 ± 8.66	101.05 ± 2.51
**Females (%)**	1265 (55%)	1507 (48%)	247 (71%)
***APOE*** ε**2 (%)**	3%	9%	13%
***APOE*** ε**4 (%)**	43%	17%	7%

*Note*: Additional information about the cohorts used is available in Methods and Supplementary Methods (in supporting information) sections. Reference to the cohorts described in this table are: Holstege et al.,^5^ van der Flier and Scheltens,^17^ Rademaker et al., ^20^ Hoogendijk et al.,^21^ Willemsen et al.,^22^ and Slot et al.^29^ Age = age at onset for AD cases, age at study inclusion for age‐matched controls, and cognitively healthy centenarians.

Abbreviations: AD, Alzheimer's disease; *APOE*, apolipoprotein E.

### 3.2 AD cases versus cognitively healthy centenarians

**FIGURE 1:**
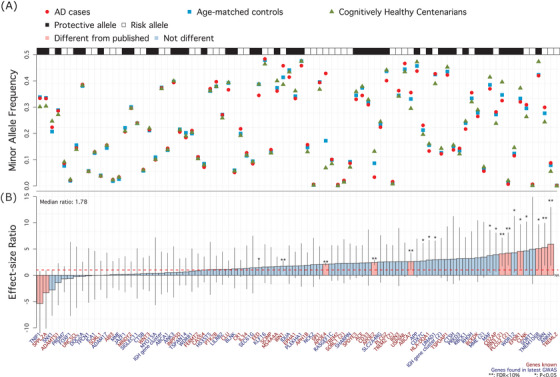
Single variant associations summary. A (top), Raw minor allele frequency in AD patients (red circles), age‐matched controls (blue squares), and cognitively healthy centenarians (green triangles). Black and white annotation squares refer to whether the plotted allele (the minor allele) was associated with an increased risk of AD (risk allele, white) or a decreased risk of AD (protective allele, black). B (bottom), Change in effect size comparing observed effect sizes (AD cases vs. cognitively healthy centenarians) to the reference effect sizes (Bellenguez et al.^10^). Blue genes refer to novel SNP–AD associations discovered by Bellenguez et al. for the first time, while red genes were known before Bellenguez et al. The dashed red line at 1 indicates the published effect size from the literature. *: *P* value of association < 0.05; **: FDR‐corrected *P* value of association < 0.05; pink bars indicate SNPs for which observed effect size is significantly different from published effect size. AD, Alzheimer's disease; FDR, false discovery rate; SNP, single nucleotide polymorphism.

Comparing AD cases to cognitively healthy centenarians, the effect size across all 86 tested SNPs increased by a median 1.78‐fold (IQR: 0.51–2.85) relative to the published effect sizes; Figure 1; Figure S2, Table S3 and Table S4 in Supplementary Tables).^10^ Overall, a relatively small difference in allele frequency may lead to a large increase in effect size (Figure 1). For 59 SNPs the change in effect size was > 1 (*P* = 3.6 × 10^−4^ based on a one‐tailed binomial test, Figure 1) and ranged from 1.07 (rs785129 near *HS3ST5* gene) to 5.91 (rs112403360 in *ANKH* gene, Table S3). Cognitively healthy centenarians did not include carriers of the rare rs60755019 (in *TREML2*), while the carriers frequency in AD cases was 0.18% and 0.14% in age‐matched controls (Table S4). For nine SNPs (in or near the genes *EPDR1, MAF, PLCG2, RIN3*, *ANKH, TMEM106B, SORT1, GRN*, and *WDR12*), the effect size was increased more than 4‐fold compared to previously published effect sizes (*change *> 4). The effect of 16 SNPs was not increased compared to the reference effect sizes (0 < *change* < 1, Figure 1 and Table S3), and the effect of 11 SNPs was opposite compared to the reference effects (*change* < 0, Figure 1, Figure S2 and Table S3). Despite the small sample size of cognitively healthy centenarians, the association for 8 out of 85 SNPs with AD reached significance after correction for multiple testing (false discovery rate [FDR] < 5%): *ANKH*, *GRN, PLCG2, RIN3, ABCA7, BIN1*, and the two *APOE* SNPs, Figure 1 and Table S3). We note that in a sensitivity analysis comparing early‐onset AD to late‐onset AD, we observed one significant association after multiple testing correction (rs7384878 in/near *ZCWPW1*, FDR < 5%, Table S5 in Supplementary Tables).

### 3.3 AD cases versus age‐matched controls

The 2281 AD patients have mainly early‐onset AD, such that they are likely enriched with risk‐increasing genetic variants relative to the predominantly late‐onset AD cases included in Bellenguez et al.^10^ Therefore, our AD dataset may explain part of the change in effect sizes observed in our AD versus cognitively healthy centenarians analysis. To investigate the contribution of the AD cases, we compared them to 3165 age‐matched controls. We observed a 1.16‐fold increased effect size relative to the published effect sizes (IQR: 0.60–1.76), which is significantly lower than the 1.78‐fold increased effect size in the comparison of AD cases versus cognitively healthy centenarians (*P* = 0.004 comparing the distributions of effect size change, Figure S3 and Table S6 in Supplementary Tables). The *change* in effect size was > 1 for 48 SNPs and ranged from 1.01 (rs73223431 near *PTK2B* gene) to 4.47 (rs141749679 near *SORT1* gene). In total, a significant association after multiple test corrections (FDR < 5%) was identified for 11 SNPs, in or near *SORT1, RHOH*, *PLCG2*, *HLA‐DQA1, EED, RIN3, APH1B, TREM2, BIN1*, and the two *APOE* SNPs, Table S6).

### 3.4 Age‐matched controls versus cognitively healthy centenarians

Next, we investigated whether the AD‐associated SNPs differentially contribute to maintaining cognitive health at old age compared to maintaining cognitive health at younger ages. For this, we compared the effect sizes of age‐matched controls to cognitively healthy centenarians and found that they were increased by a median 0.58‐fold (IQR: −0.23 to 1.45) relative to the published effect sizes in Bellenguez et al. (Figure S4 and Table S7 in Supplementary Tables).^10^ The *change* in effect size was > 2‐fold for 17 SNPs, and 1‐ to 2‐fold for 13 SNPs. The effect sizes of 29 SNPs were not increased compared to the reference effects, and the effect of 27 SNPs was opposite. Altogether, a significant association after multiple test corrections (FDR < 5%) was identified only for the two *APOE* SNPs, Table S7).

### 3.5 Polygenic risk score

**FIGURE 2:**
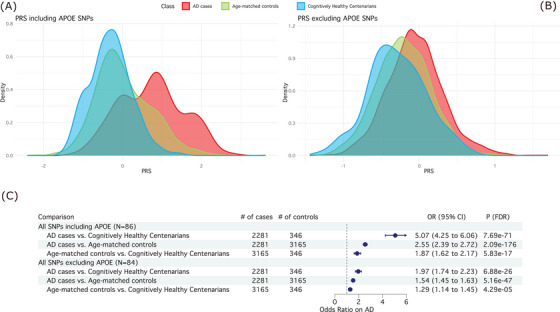
Summary of PRS analyses. A (top left), Distribution of the PRS including the two *APOE* SNPs (86 SNPs in total) in AD cases (red), age‐matched controls (blue), and cognitively healthy centenarians (green). B (top right), Distribution of the PRS excluding the two *APOE* SNPs (84 SNPs in total). C (bottom), Association statistics (OR, 95% CI, and corrected *P* value) and forest plot of the PRS including and excluding *APOE* SNPs. For the comparisons, we used logistic regression models in a pairwise manner (i.e., AD cases vs. cognitively healthy centenarians, AD cases vs. age‐matched controls, and age‐matched controls vs. cognitively healthy centenarians), controlling for population substructure. AD, Alzheimer's disease; *APOE*, apolipoprotein E; CI, confidence interval; OR, odds ratio; PRS, polygenic risk score; SNP, single nucleotide polymorphism.

We assigned two PRSs to each subject, one including the weighted effect of all 86 SNPs, and a second excluding the effect of the two *APOE* SNPs (Figure 2). Then, we compared the distribution of the PRSs among AD cases, age‐matched controls, and cognitively healthy centenarians (Figure 2 and Table S8 in Supplementary Tables). In all comparisons, the PRSs in AD cases was significantly higher. AD patients versus age‐matched controls, excluding the two *APOE* SNPs: odds ratio [OR] = 1.54, 95% confidence interval [CI] = [1.45–1.63], *P* = 1.55 × 10^−47^; including *APOE* SNPs: OR = 2.55, 95% CI = [2.39–2.72], *P* = 2.09 × 10^−176^). AD patients versus cognitively healthy centenarians, excluding the two *APOE* SNPs: OR = 1.97, 95% CI = [1.74–2.23], *P* = 2.75 × 10^−26^; including *APOE* SNPs, OR = 5.07, 95% CI = [4.25–6.06], *P* = 1.54 × 10^−71^). We found a significantly lower PRS in centenarians compared to age‐matched controls, excluding *APOE* SNPs: OR = 0.77, 95% CI = [0.69–0.88], *P* = 2.57 × 10^−5^; including *APOE* SNPs, OR = 0.53, 95% CI = [0.46–0.62], *P* = 2.92 × 10^−17^. Notably, all analyses remained significant after splitting by sex. The only exception was that the PRS between cognitively healthy centenarian males and healthy control males (PRS without *APOE*) lost its statistical significance, likely attributable to the limited number of male centenarians (*N* = 99, OR = 1.14, 95% CI = [0.92–1.41], *P *= 2.44 × 10^‐1^; Table S8). Also, in this sample, the distribution of PRSs of the early‐onset AD cases was not different from the late‐onset AD cases (*P *> 0.05, Table S8).

### 3.6 The contribution of a centenarian

**FIGURE 3:**
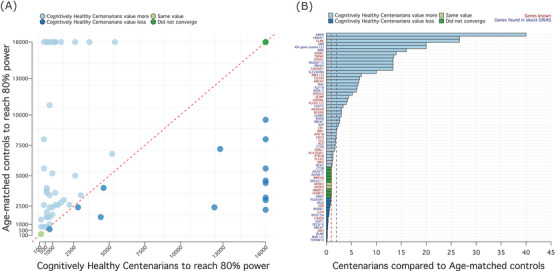
Relationship between cognitively healthy centenarians and age‐matched controls. A (left), Number of individuals (age‐matched controls on the y axis and centenarians on x axis) necessary to achieve 80% power for a SNP association at *P* = 0.05, assuming 8000 AD cases. We restricted this analysis to common variants (MAF > 1%) with expected direction of effect in our comparisons (*N* = 67 SNPs, see Methods). Note that, for this reason, some variants enriched in cognitively healthy centenarians such as rs13237518 (*TMEM106B*) and rs13237518 (*SORT1*) could not be represented here. Each dot represents a SNP: dark green dots identify the eight SNPs that did not converge using both age‐matched controls and centenarians (i.e., the power did not reach 80%). Light green dots indicate the two *APOE* SNPs, for which *N* = 200 individuals (age‐matched controls and centenarians) were enough to guarantee 80% power. Light blue dots identify SNPs for which the number of cognitively healthy centenarians (to achieve 80% power) was lower than the number of age‐matched controls. Of these, *N* = 13 SNPs did not converge using age‐matched controls. Conversely, dark blue dots identify SNPs for which the number of age‐matched controls was lower than the number of cognitively healthy centenarians. Of these, *N* = 8 SNPs did not converge using cognitively healthy centenarians. B (right), Ratio between the number of age‐matched controls and the number of cognitively healthy centenarians, for each SNP. Color code is the same as (A). SNPs larger than the blue dotted line (*N* = 31, ratio > 2) were used for functional annotation and gene‐set enrichment analysis. AD, Alzheimer's disease; *APOE*, apolipoprotein E; GWAS, genome‐wide association study; MAF, minor allele frequency; SNP, single nucleotide polymorphism.

In a simulation, we estimated the number of age‐matched controls and cognitively healthy centenarians required to reach 80% power to find an association at *P* = 0.05, we used a subset of 67 common SNPs for which the direction of effect in our analyses matched that of Bellenguez et al. (see section 2.5: The contribution of a centenarian; Table S3, and Table S9 in Supplementary Tables). For eight SNPs, a total of 16,000 controls did not guarantee the power of 80% (i.e., no convergence) using both age‐matched controls and cognitively healthy centenarians, which is likely due to the small effect sizes associated with these SNPs (Figure 3A and Table S9). For the remaining 59 SNPs, an association at *P* = 0.05 (convergence) was observed comparing 8000 AD cases with on average 6183 ± 5680 age‐matched controls (median = 3600, IQR = 2300–8800) or 3745 ± 5436 cognitively healthy centenarians (median = 1200, IQR = 600–3300; Figure 3 and Table S9). On average, based on 59 AD SNPs, and specifically within our cohort of individuals, the power of a single cognitively healthy centenarian in a GWAS of AD is equivalent to that of 5.86 typical age‐matched controls (median = 2.4, IQR = [1.00–6.58], Figure 3 and Table S9).

### 3.7 Functional implications

**FIGURE 4:**
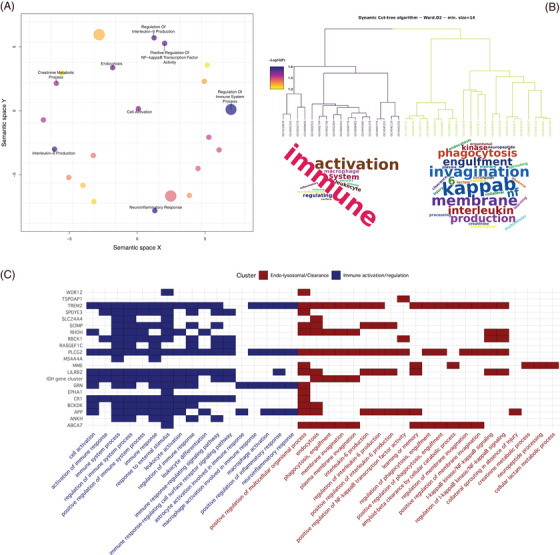
Functional annotation of SNPs with the largest effect in cognitively healthy centenarians. The figure shows the result of the functional annotation of 31 SNPs for which the number of cognitively healthy centenarians required to achieve 80% power was at least half of the number of age‐matched controls required to achieve the same power. Functional annotation analysis was performed using snpXplorer.^27^ A, Result of the gene‐set enrichment analysis followed by REVIGO analysis, which clusters enriched pathways based on a semantic similarity measure. B, Dendrogram of the main enriched pathways along with their cluster (branches color code for cluster assignment) and word clouds showing the main terms enriched in the underlying pathways. C, Mapping between significant pathways (x axis), AD‐associated SNPs (y axis, labeled with the name of the gene as provided by Bellenguez et al.^10^), and the relative gene‐set enrichment cluster. AD, Alzheimer's disease; SNP, single nucleotide polymorphism.

We then functionally annotated and performed gene‐set enrichment analysis using 31 SNPs for which the power of a single centenarian was > 2‐fold increased than age‐matched controls (Figure 3B). Of 31 SNPs, only 2 were coding (rs143332484 in *TREM2* and rs72824905 in *PLCG2*); 23 were annotated to their likely affected gene(s) using eQTL, sQTL, and Combined Annotation Dependent Depletion (CADD) information; and 6 SNPs were annotated solely based on their genomic position (Table S10 in Supplementary Tables). The resulting genes were used as input for gene‐set enrichment analysis. After clustering the enriched Gene Ontology (GO) terms based on a semantic similarity measure, we found two clusters of pathways, pointing toward the immune system and endo‐lysosomal trafficking (Figure 4A‐B and Table S11 in Supplementary Tables). The immune system cluster of pathways included activation and regulation of immune response (genes *CR1, MS4A6A, IGH‐cluster, RIN3, KAT8, GRN, SCIMP, RBCK1, APP, RHOH, OTULIN, MAPK9, PLCG2*, and *TREM2*), leukocyte activation and differentiation (genes *CD55, CR1, IGH‐cluster, APP, GRN, PLCG2*, and *TREM2*), macrophage activation (genes *GRN, APP, PLCG2*, and *TREM2*), and neuroinflammatory response (genes *GRN, LILRA5, PLCG2, KAT8*, and *TREM2*). The endo‐lysosomal trafficking cluster of pathways included marked immunological aspects: endocytosis and phagocytosis (genes *IGH‐cluster, RIN3, ABCA7, LILRB4, APP, RHOH, PLCG2*, and *TREM2*), interleukin‐6 metabolism (genes *SCIMP, LILRA5, APP, PLCG2*, and *TREM2*), and amyloid clearance (genes *ABCA7, MME, APP*, and *TREM2*) (Figure 4C, Table S11). We compared these clusters to five clusters from a previous study including all AD‐associated SNPs.^27^ A significant overlap was found only between the *endolysosomal trafficking* cluster (this analysis) and (1) the amyloid clearance cluster (previous study, chi‐square *P* = 3.38 × 10^−5^), and (2) immune trafficking and migration cluster (previous study, chi‐square *P* = 2.07 × 10^−4^). Conversely, no significant overlap was found regarding clusters of pathways pointing to activation of immune response (*P* = 0.49), organization and metabolic processes, and amyloid beta (Aβ) and tau formation.

## 4 DISCUSSION

Based on common AD‐associated SNPs as identified by GWAS, self‐reported cognitively healthy centenarians from the 100‐plus Study are genetically protected against AD. Cognitively healthy centenarians have a lower frequency of almost all risk‐increasing alleles and a higher frequency of protective alleles, which indicates that maintaining cognitive health depends on having an advantageous function across all genetically associated mechanisms. However, the centenarians are most strongly depleted with the risk alleles in *ANKH, GRN*, and *SORT1*, and most strongly enriched with the protective alleles in *TMEM106B*, *EPDR1*, *PLCG2* (rs72824905), and *RIN3* (rs12590654). For these alleles, the effect sizes were > 4‐fold increased comparing AD cases to cognitively healthy centenarians rather than age‐matched controls. Together, our findings suggest that prolonged cognitive health depends on maintaining specific aspects of the endolysosomal and immune system, and on the resistance of accumulating neuropathology.

The centenarians had a significantly lower PRS for AD compared to middle‐aged healthy individuals, both including and excluding the effect of the two *APOE* alleles. The effect size across risk alleles was increased by an average 1.78‐fold comparing AD cases to cognitively healthy centenarians as controls rather than age‐matched controls. These effects were confirmed in both males and females. However, the increase in effect size concentrates on specific alleles, indicating that prolonged cognitive health especially depends on the maintenance of the associated cellular processes.

Cognitively healthy centenarians are most strongly enriched with the protective allele of the *ANKH* gene (rs112403360), which is associated with hippocampal sclerosis and Braak neurofibrillary tangles stages.^30^ Impairment of the *ANKH* gene leads to excessive mineralization, including calcification of arteries leading to joint pain, arthritis, atherosclerosis, and diabetes.^31^, ^32^ Together, this suggests that the prolonged cognitive health in centenarians may be supported by maintained vasculature and low pathology load in the brain.

Furthermore, it is intriguing that the protective alleles of the *GRN‐*, *TMEM106B‐*, and the *SORT1*‐associated loci are among the strongest enriched in cognitively healthy centenarians, as these three genes all contribute to endolysosomal trafficking.^33^, ^34^, ^35^ It is notable that these loci were previously identified in context of frontotemporal lobar degeneration (FTLD) risk.^36^, ^37^ This might suggest that these FTLD risk alleles also influence the risk of AD; that some AD patients may have FTLD as a comorbidity; or that FTLD patients were misdiagnosed as AD patients, influencing the GWAS.^30^ Regardless of rationale, the strong enrichment of these three alleles underlines the importance of a functional endolysosomal trafficking mechanism in maintained cognitive health during aging. This is further supported by a strong enrichment of the protective allele of *RIN3* (rs12590654 and rs7401792), the function of which is also associated with endolysosomal function and axonal trafficking.^38^

*EPDR1* (mammalian ependymin‐related protein 1) is a transmembrane protein that plays a crucial role in adhesion of neural cells.^39^ Although its role in AD is currently not clear, *EPDR1* was shown to be downregulated in AD patients compared to controls,^40^ and has been implicated in dopaminergic regulation of neurogenesis and neuroendocrine function in goldfish.^41^ While speculative, our finding that cognitively healthy centenarians are enriched with a protective *EPDR1* allele may confirm a role for prolonged neurogenesis in maintaining cognitive health.^42^

Protective alleles in genes modulating immune and neuroinflammatory response (*PLCG2, CR1, TREM2, OTULIN, MS4A‐cluster*) were strongly enriched in cognitively healthy centenarians, suggesting that maintaining an efficient regulation of neuro‐immune response during aging is an important aspect of cognitive health. Notably, the protective coding SNP rs72824905, leading to the gain‐of‐function p.P522R substitution in *PLCG2*, provides proof of concept that only a limited increase in immune activation translates to a beneficial effect, as stronger gain‐of‐function mutations in *PLCG2* (e.g., p.S707Y and p.L848P) are associated with autoimmune disorders such as PLCγ2‐associated antibody deficiency and immune dysregulation syndrome (PLAID) and autoinflammation, antibody deficiency, and immune dysregulation syndrome (APLAID).^13^, ^43^, ^44^

*TREM2* is well known to be involved in microglial activation and phagocytosis in the same pathway as *PLCG2*. The protective allele of the rs75932628 coding SNP in *TREM2* (i.e., the arginine at residue 74), was enriched in the centenarians and was shown to increase microglial activation and expression of proinflammatory cytokines.^45^ Altogether, a slightly more responsive immune and neuroinflammatory response in cognitively healthy centenarians seems to better cope with the physiological accumulation of pathology over time and promote a long‐term maintenance of cognitive health.^46^

The protective alleles of SNPs near *ABCA7* (rs12151021)*, SORL1* (rs74685827)*, APP* (rs2154481), and *APOE* (rs429358 and rs7412) were all enriched in cognitively healthy centenarians. These genes are involved in immune–lipid signaling pathways that lead to the clearance of amyloid peptides in the brain.^47^ Specifically, the *ABCA7* gene is involved in Aβ processing and clearance, while the *SORL1* gene codes for a retromer receptor involved in the trafficking of amyloid precursor protein (APP), thereby preventing Aβ secretion.^13^, ^48^ Interestingly, in the brains donated by cognitively healthy centenarians we observed Aβ deposits across many regions; however, the load of Aβ neuropathology remained limited.^49^ This suggests that enrichment of protective alleles may support the resistance of the accumulation of amyloid pathology.

Finally, we expect that the genetically driven enhancements of conserved molecular mechanisms will have limited functional effects, as impactful changes are likely to have damaging effects. This is exemplified by the limited functional effects of strongly protective coding variants in *PLCG2, APP*, and *APOE*.^13^, ^46^, ^50^, ^51^

While the main aim of this study was to identify the AD‐associated genetic loci that most prominently associated with escaping AD, our study also suggests that genetic comparisons of diseased individuals to those who are resilient to the disease maximize the identified effect sizes. The comparison of AD patients and age‐matched controls yielded effect sizes comparable to published effect sizes, highlighting that the observed increased effects were mostly due to the cognitively healthy centenarians. In fact, 84% (37/44) of the SNPs that were associated with AD for the first time in Bellenguez et al. had the same direction of effects, despite their very small effect sizes. We were able to replicate the association of 8 SNPs at FDR < 5%, while a comparison of these AD cases with more than 10 times the number of age‐matched controls allows for the replication of 11 SNPs. Together, we estimated that the contribution of one centenarian to a case–control analysis is equivalent to on average six age‐matched controls. While this highlights the power of analyzing extreme phenotypes, we acknowledge that assembling a sufficiently large cohort of cognitively healthy centenarians for the discovery of novel disease loci in a case–control comparison is challenging.^5^ Furthermore, because maintained cognitive health concurs with extreme longevity in our centenarians, the effect sizes on pure AD risk of such newly identified loci would have to be determined in a (targeted) age‐matched comparison between AD cases and age‐matched controls. Indeed, prior analyses have demonstrated that cognitively healthy centenarians carry an abundance of longevity‐related genetic variations, some of which might even alleviate the adverse effect of *APOE* ε4 allele.^42^, ^51^ We acknowledge that, given the association between maintained cognitive health and maintained physical health,^52^ genetic differences associated with resilience against AD may also be representative of overall survival until extreme age. However, the 86 SNPs studied here were all discovered in an age‐matched GWAS of AD and, except for *APOE*‐ and *HLA*‐associated SNPs, these are not detected by previous GWASs of survival, longevity, and/or other age‐related diseases.^14^, ^53^, ^54^, ^55^, ^56^ This suggests that the tested alleles may be in large part representative of resilience to AD rather than overall decline, and that the differential effect sizes when using cognitively healthy centenarians as controls points toward the most important mechanisms associated with escaping AD and other dementias until old age. In addition, to disentangle the genetics underlying AD resilience, it would be ideal to compare cognitively healthy centenarians to centenarians who are affected with AD, representing an estimated 75% of all centenarians in the population.^57^ However, ethical considerations precluded the inclusion of centenarians affected with AD in the 100‐plus Study. Therefore, for this analysis, we had to refrain to comparisons to unaffected and affected younger individuals.

Our study was conducted in a genetically homogeneous population: cognitively healthy centenarians as well as AD cases and age‐matched controls are all from the same Dutch (White) population, and we are aware that genetic associations with AD differ among individuals from different ancestries,^58^ which likely extends to the genetics associated with the long‐term maintenance of cognitive health. We acknowledge that AD patients, age‐matched controls, and cognitively healthy centenarians were from different studies, each with their own inclusion criteria: individuals with subjective cognitive decline (included as age‐matched controls) and the AD patients presented at the clinic with complaints, while the participants of the 100‐plus and LASA studies were actively approached for study inclusion. Therefore, we cannot exclude that comparisons were affected by inclusion biases introduced by differences between individuals willing to contribute to research and those seeking care for clinical complaints. Last, we acknowledge that part of the individuals used in this study was also included in the GWAS study we used as a reference. However, these individuals represent < 2% of all AD cases included in the GWAS, and < 0.5% of all controls included, a negligible fraction.

In summary, we find that cognitively healthy centenarians are genetically protected against AD and that the alleles with the largest effects are involved in sustaining specific aspects of the immune and endolysosomal systems, which may prevent accumulation of amyloid and other neuropathological hallmarks of AD.

## CONFLICT OF INTEREST STATEMENT

The authors declare no conflicts of interest. Author disclosures are available in the supporting information.

## CONSENT STATEMENT

The medical ethics committee of the Amsterdam UMC approved all studies. All participants and/or their legal representatives provided written informed consent for participation in clinical and genetic studies.

## Supporting information

Supplementary Methods and Figures

Supplementary Tables

Disclosure Form
