# Supplementary material for: Cognitively healthy centenarians are genetically protected against Alzheimer's disease
Source: Alzheimers Dement. 2024 Apr 18;20(6):3864–75. doi: 10.1002/alz.13810 (PMC11180929; doi:10.1002/alz.13810)
Supplement: Supplementary file 1 — Supplementary Methods and Figures [file ALZ-20-3864-s001.docx]

**Supplementary Methods**

*Additional information about the cohort used*

The Netherlands Brain Bank (NBB) is a non-profit organization that collects human brain tissue of donors who live in the Netherlands with a variety of neurological and psychiatric disorders, but also of non-diseased donors. Brain tissues, together with an anonymized summary of a donor’s medical record, is sent to scientific researchers worldwide, with the ultimate aim of increasing our understanding of the human brain and to develop therapies for neurological and psychiatric diseases. To date, the NBB has provided researchers with brain tissue from 1000 patients with Alzheimer disease, 285 with Parkinson disease, 240 with multiple sclerosis, and 1260 healthy controls without brain diseases. [1]

The Longitudinal Aging Study Amsterdam (LASA) is a prospective cohort study to determine predictors and consequences of aging. Since 1992, LASA has studied the physical, emotional, cognitive and social functioning of the elderly in the Netherlands. Data collection started in 1992-1993 based on municipal registries, among a cohort of respondents aged 55-84 years old (cohort 1). An additional cohort of respondents aged 55-64 years (cohort 2) was included from the same sampling frame and was measured for the first time exactly ten years after the original baseline measurement, in 2002-2003. In 2012-2013, a third cohort study (cohort 3) was initiated with new respondents aged 55-64 years from the same sampling frame. Measurement cycles usually consist of a main interview, and a medical interview with clinical measurements. LASA study collects a wide range of individual information including, socio-economical status, neuropsychological measures, and biological material and measures. [2] At the time of inclusion in this study, all individuals were cognitively healthy.

The SCIENCe is a prospective cohort study including consecutive patients with subjective cognitive decline (SCD) presenting at the Alzheimer Center of the VU University Medical Center Amsterdam. [3] Inclusion criteria for the SCIENCe are a diagnosis of SCD and age ≥ 45 years. Exclusion criteria are mild cognitive impairment (MCI), dementia, major psychiatric disorder (i.e., current depression, personality disorders, schizophrenia), neurological diseases known to cause memory complaints (i.e., Parkinson’s disease, epilepsy), HIV, abuse of alcohol or other substances, and language barrier. [4]

*Genotyping an Imputation*

Genetic variants were determined by standard genotyping and imputation methods. Briefly, we genotyped all individuals using the Illumina Global Screening Array and applied established quality control methods. We used high-quality genotypes in all individuals (individual call rate >99%, variant call rate >99%), individuals with sex mismatches were excluded and departure from Hardy-Weinberg equilibrium was considered significant at p<1x10^-6^. Genotypes were then lifted over to GRCh38 and prepared for imputation using provided scripts (HRC-1000G-check-bim.pl) specifying TOPMED as reference panel. [5] This script compares variant ID, strand, and allele frequencies to the TOPMED reference panel (version r2, N=194,512 haplotypes from N=97,256 individuals). [6] Finally, all variants were submitted to the Michigan Imputation server (<https://imputation.biodatacatalyst.nhlbi.nih.gov/>). The server uses EAGLE (v2.4) to phase data and Minimac4 to perform genotype imputation to the reference panel (version r2). [7, 8]

*Change in effect size*

The *change* in effect size was calculated using the same approach adopted in [9]. Briefly, the *change* refers to the ratio between the reference effect sizes and the observed effect sizes when comparing (i) AD cases with centenarians, (ii) AD cases with age-matched healthy controls, and (iii) healthy controls with centenarians. To calculate the observed effect sizes, we used logistic regression models correcting for population stratification (principal components 1-5). We calculated effect sizes and odds ratios relative to the least frequent allele assuming additive genetic effects, and estimated 95% confidence intervals. The change in effect size is then, for each SNP, the ratio between the observed effect size in each comparison and the reference effect size. When the change equals 1, the observed and reference effect sizes are the same; when the change is larger than 1, the observed effect size is larger than the reference effect; when the change is smaller than 1, yet positive, the reference effect size is larger than the observed effect; when the change is negative, the directions of the observed and the reference effect sizes are opposite.

**References**

1. Rademaker MC, de Lange GM, Palmen SJMC. The Netherlands Brain Bank for Psychiatry. Handbook of Clinical Neurology, vol. 150, Elsevier; 2018. p. 3–16.

2. Huisman M, Poppelaars J, van der Horst M, Beekman AT, Brug J, van Tilburg TG, et al. Cohort Profile: The Longitudinal Aging Study Amsterdam. International Journal of Epidemiology. 2011;40:868–876.

3. van der Flier WM, Scheltens P. Amsterdam Dementia Cohort: Performing Research to Optimize Care. Journal of Alzheimer’s Disease. 2018;62:1091–1111.

4. Slot RER, Verfaillie SCJ, Overbeek JM, Timmers T, Wesselman LMP, Teunissen CE, et al. Subjective Cognitive Impairment Cohort (SCIENCe): study design and first results. Alzheimer’s Research & Therapy. 2018;10.

5. McCarthy S, Das S, Kretzschmar W, Delaneau O, Wood AR, Teumer A, et al. A reference panel of 64,976 haplotypes for genotype imputation. Nature Genetics. 2016;48:1279–1283.

6. Taliun D, Harris DN, Kessler MD, Carlson J, Szpiech ZA, Torres R, et al. Sequencing of 53,831 diverse genomes from the NHLBI TOPMed Program. Nature. 2021;590:290–299.

7. Das S, Forer L, Schönherr S, Sidore C, Locke AE, Kwong A, et al. Next-generation genotype imputation service and methods. Nature Genetics. 2016;48:1284–1287.

8. Fuchsberger C, Abecasis GR, Hinds DA. minimac2: faster genotype imputation. Bioinformatics. 2015;31:782–784.

9. Tesi N, van der Lee SJ, Hulsman M, Jansen IE, Stringa N, van Schoor N, et al. Centenarian controls increase variant effect sizes by an average twofold in an extreme case–extreme control analysis of Alzheimer’s disease. European Journal of Human Genetics. 2018. September 2018. https://doi.org/10.1038/s41431-018-0273-5.

**Supplementary Figures**

***Figure S1: Age distributions of the individuals included in our study, separated by the sample type.***

*****Figure S2*: *Single variant associations summary comparing AD cases and cognitively healthy centenarians.*** The figure shows the SNP effect sizes relative to the comparison of AD cases and cognitively healthy centenarians (blue bars) and as reported in the reference GWAS (red bars).

 **Figure S3: Single variant associations summary comparing AD cases and age-matched controls.** Figure A shows the SNP effect sizes relative to the comparison of AD cases and age-matched controls (blue bars) and as reported in the reference GWAS (red bars). Figure B shows the change in effect size when comparing observed effect sizes (AD cases vs. age-matched controls) to the reference effect sizes. The dashed red line at 1 indicates the published effect size from the literature. Negative bars refer to a different direction of effect between the GWAS we used as a reference and our Study. Bars lower than 1 (yet positive), refer to SNP whole effect-size from the GWAS we used as a reference was larger than the observed effect-size.

 **Figure S4: Single variant associations summary comparing age-matched controls and cognitively healthy centenarians.** Figure A shows the SNP effect sizes relative to the comparison of age-matched controls and cognitively healthy centenarians (blue bars) and as reported in the reference GWAS (red bars). Figure B shows the change in effect size when comparing observed effect sizes (Age-matched controls vs. Cognitively Healthy Centenarians) to the reference effect sizes. The dashed red line at 1 indicates the published effect size from the literature. Only the association of the 2 SNPs in *APOE* remained significant after correcting for multiple tests. Negative bars refer to a different direction of effect between the GWAS we used as a reference and our Study. Bars lower than 1 (yet positive), refer to SNP whole effect-size from the GWAS we used as a reference was larger than the observed effect-size.
